# Implementing palliative reasoning in multiprofessional palliative care: A participatory action research in the Netherlands

**DOI:** 10.1371/journal.pone.0354089

**Published:** 2026-08-28

**Authors:** Katrin Kochems, Saskia C. C. M. Teunissen, Ginette M. Hesselmann, Marjolein Verkammen, Gon Uyttewaal, Karin Kalthoff, Alexander de Graeff, Everlien de Graaf

**Affiliations:** 1 Center of Expertise in Palliative Care, Julius Center for Health Sciences and Primary Care, University Medical Center Utrecht, Utrecht, The Netherlands; 2 Cancer Center, University Medical Center Utrecht, Utrecht, The Netherlands; 3 Dutch Institute of Palliative Care, Utrecht, The Netherlands; 4 Opella, Ede, Gelderland, The Netherlands; 5 Zorgbelang inclusief, Arnhem, Gelderland, The Netherlands; 6 Department of Medical Oncology, University Medical Center Utrecht, Academic Hospice Demeter, De Bilt, Utrecht, The Netherlands; Queensland University of Technology - QUT: Queensland University of Technology, AUSTRALIA

## Abstract

**Context:**

In the Netherlands, two interventions exist to support healthcare professionals in assessing palliative care needs and providing multidimensional generalist palliative care. Separate use hindered communication, interprofessional collaboration, and multidimensional care. To address this, the two existing interventions were integrated into a single multiprofessional intervention, Palliative Reasoning. The Palliative Reasoning Working Method (PRWM) was developed to support its implementation in daily practice.

**Objectives:**

To iteratively adapt and implement the PRWM in primary care, hospice, and nursing home settings, and to explore determinants, implementation strategies, implementation outcomes, and healthcare professionals perceived impact.

**Methods:**

A multi-method participatory action research with three iterative cycles was conducted between October 2020 and January 2022 in Dutch primary care, hospice, and nursing home settings. Earlier phases included practice analysis and co-design of the PRWM. In this phase, PRWM was adapted and implemented within 20 multiprofessional teams. Primary outcomes were adaptation and implementation (acceptability, adoption, feasibility, fidelity, and sustainability); secondary outcome concerned healthcare professionals’ perceived impact. FRAME guided adaptation documentation. A logic model, informed by CFIR, ERIC and COM-B, explored implementation outcomes. Qualitative and quantitative data were analyzed deductively and descriptively, respectively.

**Results:**

Fourteen content adaptations and seven supporting materials were developed. A logic model depicted key determinants, strategies, mechanisms, and outcomes of implementation. The PRWM was perceived as feasible and acceptable, improving recognition of palliative care needs, person-centered care, nursing empowerment, and team collaboration. Implementation proceeded largely as planned, adapted to local contexts, and continued in most teams. Integration into electronic health records and organizational embedding were crucial for sustainability. Healthcare professionals reported increased knowledge, motivation, and leadership skills.

**Conclusion:**

PRWM shows promise for embedding Palliative Reasoning into routine palliative care across primary, hospice, and nursing home settings, strengthening proactive, person-centered, and collaborative practice, especially through nursing empowerment. Sustainable implementation requires behavioral-change strategies and supportive organizational and digital infrastructure.

## Introduction

In the Netherlands, incorporation of a palliative care approach is needed in primary care and nursing home care, as 38% of individuals with palliative care needs die at home, and 28% in nursing homes [1]. Palliative care is provided through an interprofessional collaboration of generalist healthcare professionals (HCPs), mostly general practitioners, nursing home physicians and nurses [2]. When required, specialist HCPs in palliative care with additional training in palliative care support generalists in managing complex situations through consultation [3]. A palliative care approach prevents and relieves suffering by means of early identification, assessment and management of physical, psychological symptoms, social and spiritual concerns [2]. Based on this approach, palliative care is provided in accordance with the values, wishes, and needs of patients and their relatives [2].

Analysis of current practice in primary care and nursing homes identified several barriers to the provision of palliative care: difficulties in estimating life expectancy, limited use of assessment tools to systematically identify, monitor and assess patients’ symptoms and concerns in the palliative phase, insufficient expert consultation, limited multiprofessional consultations, and inadequate documentation in electronic health records, particularly duplicate documentation and unclear responsibilities [4]. These barriers may arise from underlying mechanisms such as prognostic uncertainty, insufficient knowledge and training of HCPs, the absence of a standardized working method, and inadequate organization of collaboration and healthcare systems, including non-standardized electronic health records and limited possibilities for interprofessional collaboration. Consequently, these barriers increase the risk of delayed or missed identification of patients in the palliative phase, insufficient multidimensional care, poor information transfer, and fragmented care delivery. In addition, a qualitative study among patients with an estimated life expectancy of less than one year who were receiving care at home or in nursing homes, and their relatives, showed that patients and relatives considered it important to be seen as a unique person, to receive honest and clear information, and having care organized to ensure continuity [5]. A clear and structured approach within the multiprofessional team is essential, which is centered on the patient [4,5].

**Fig 1:**
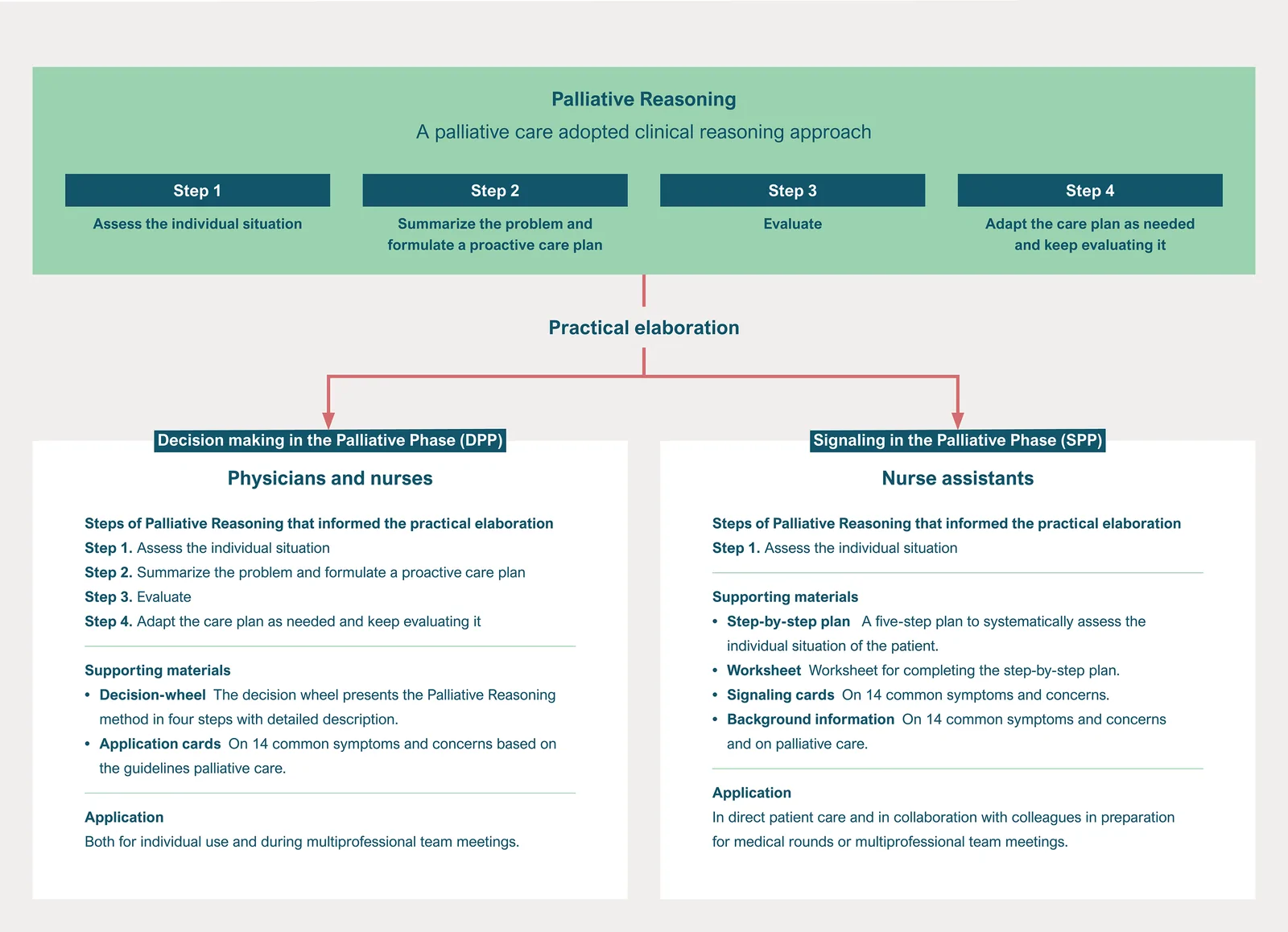
Palliative Reasoning methodology, Decision making and Signaling in the palliative phase.

To support HCPs in multidimensional generalist palliative care, a structured approach known as Palliative Reasoning has been developed in the Netherlands. Palliative Reasoning is a clinical reasoning model specifically adapted for palliative care to optimize systematic attention for all dimensions following an iterative process: (1) assess the individual situation; (2) summarize the problem and its causes and formulate a proactive care plan; (3) evaluate; and (4) adapt the care plan as needed and keep evaluating [6–9]. To bring Palliative Reasoning into clinical practice, two interventions were developed in succession for different user groups (Fig 1): 1) Decision making in the Palliative Phase, developed in 2008 for physicians and nurses to support the application of Dutch palliative care guidelines [6,10] and; 2) Signaling in the Palliative Phase, developed in 2012 to assist nurse assistants in identifying, interpreting and articulating problems that most commonly occur in the palliative phase. Signaling in the Palliative Phase enables nurse assistants to better engage in conversations with patients and their relatives and to prepare for or contribute to multiprofessional consultations [11]. Although both interventions are based on Palliative Reasoning, they are often applied separately which hampers collaboration, communication and multidimensional symptom management.

As multidimensionality and interprofessional collaboration are core principles of palliative care [2], the existing use of both interventions was considered insufficiently efficient. Therefore, the KWASA study *(Quality of care. Collaboration in signaling and decision making in the palliative phase)* was initiated to integrate Decision making in the Palliative Phase and Signaling in the Palliative Phase into a single intervention for multiprofessional users and to implement this intervention in primary care and nursing home care practice. The Implementation of Change Model served as a framework for our study [12]. In an earlier study phase, the two existing interventions were merged into a single integrated intervention, Palliative Reasoning, for multiprofessional teams.

The purpose of this study was to iteratively adapt the integrated intervention for multiprofessional teams and implement it within multiprofessional teams in primary care and nursing home settings.

## Methods

### Design

A multiple-method study was conducted between October 2020 and January 2022 in primary care, hospice, and nursing home settings in the Netherlands using a Participatory Action Research (PAR) approach. A constructivist paradigm primarily guided the study, complemented by elements of a pragmatic paradigm to adapt and implement the integrated multiprofessional working method. The study followed the action research spiral model by Kemmis and McTaggart [13] (Table 1).

**Table 1:** Kemmis and McTaggart’s action research spiral.

A spiral of self-reflective cycles of:
1. **Planning** a change;
2. **Acting** and **observing** the process and consequences of the change;
3. **Reflecting** on these processes and consequences;
4. **Replanning.**

### Setting and participants

Homecare organizations and nursing homes in the central region of the Netherlands were recruited by means of convenience sampling using the researchers’ network. Participatory teams were eligible for inclusion if organizational management expressed willingness to participate in the study, and at least one contact person could be assigned to facilitate communication and coordination.

### Intervention

**Fig 2:**
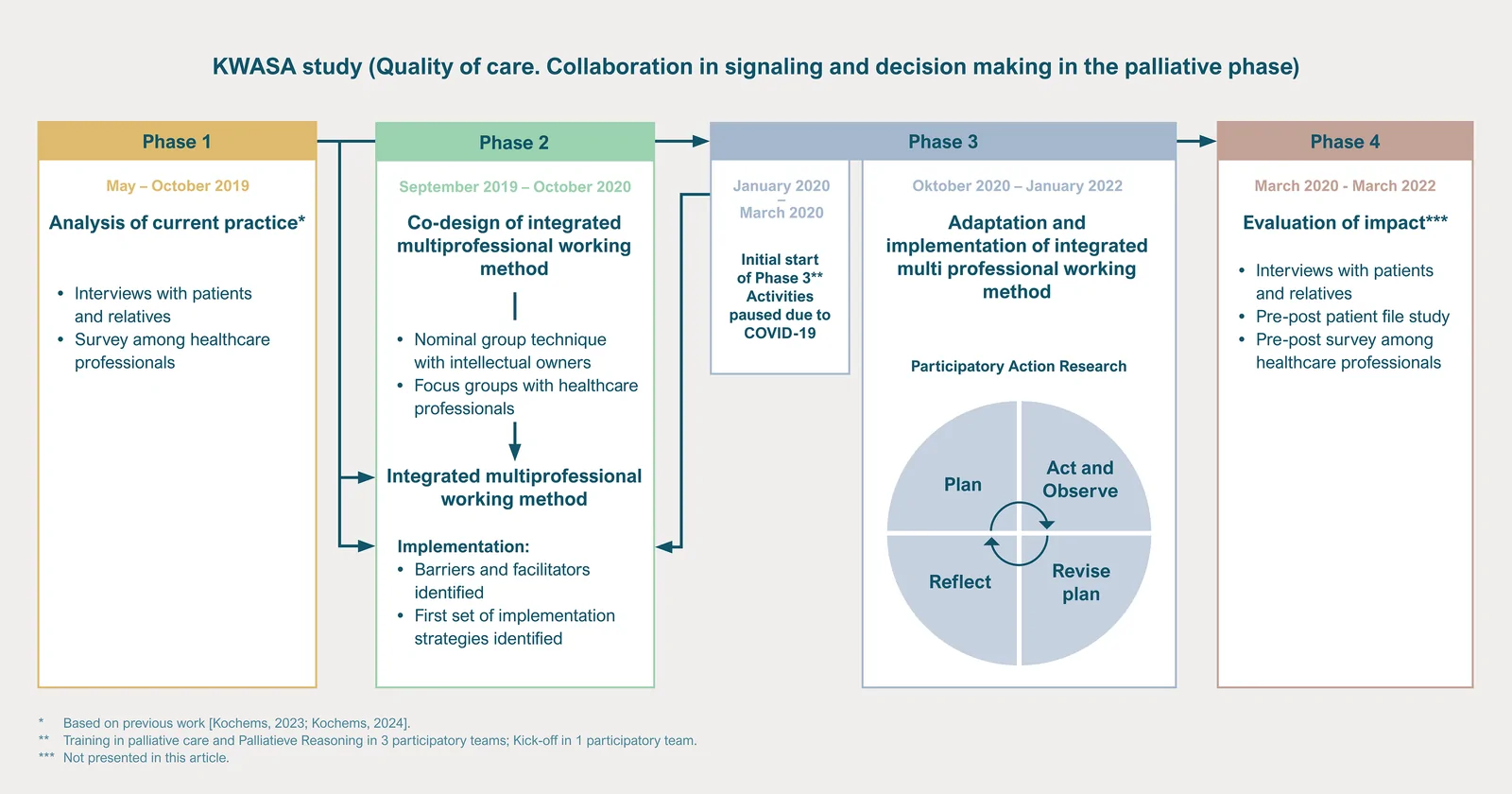
Overview of the study phases.

Prior to the implementation phase, an analysis of current practice and co-design of the integrated multiprofessional working method took place between May 2019 and October 2020 (Fig 2). The co-design phase was informed by stakeholder involvement using a nominal group technique with the intellectual owners of the existing interventions (DPP and SPP), and four focus groups with HCPs from included homecare organizations and nursing homes.

The analysis and co-design phases resulted in the integration of DPP and SPP into a single multiprofessional intervention, named Palliative Reasoning. To facilitate the implementation of Palliative Reasoning in daily practice, the **Palliative Reasoning Working Method (PRWM)** was developed. The first version of the PRWM consisted of:

1. Patient identification
2. Palliative Reasoning

First, patients with possible palliative care needs should be identified using the “Surprise Question”: “Would I be surprised if this patient died within the next year?” [14]. If the answer is “No,” the patient may have palliative care needs, and the four steps of Palliative Reasoning can subsequently be applied (Fig 1).

#### Implementation phase.

During this current study, the PRWM was adapted and implemented through three action research cycles over a period of approximately one year. Each cycle comprised planning, acting and observing, reflecting, and revising [13], and was conducted within the action group of each participatory team. The action groups consisted of HCPs and researchers working collaboratively throughout the process.

The analysis and co-design phases identified barriers and facilitators that informed the selection of implementation strategies. In addition, experiences from the initial implementation activities further informed the development of these strategies (Fig 2). Following the first focus group in the co-design phase, the implementation phase was initiated, with educational sessions conducted in three participatory teams and the first action research cycle started in one team (January–March 2020). In March 2020, the first COVID-19 outbreak occurred, and all project activities had to be put on hold.

Prior to the action cycles, participatory teams received a two-hour training session on palliative care and Palliative Reasoning. Cycle 1 began with a tailored kickoff meeting, facilitated by the researcher, to establish team-specific goals. At the end of each cycle, a two-hour reflection meeting was held and facilitated by the research team and contact persons. During cycle 2, plans for subsequent implementation activities were developed, while cycle 3 focused on sustainability beyond the implementation period. In addition, cycle 2 included training on assessment tools, and cycle 3 included an additional training session on Palliative Reasoning.

Furthermore, learning collaboratives, in the form of multiprofessional patient discussions, were established to support the stepwise implementation of the PRWM in daily practice and were conducted throughout the implementation phase. For identified local champions, a train-the-trainer strategy was implemented, in which experienced coaches trained and coached the champions.

### Outcomes

The two primary outcomes of this study comprised (1) the **adaptation** and (2) the **implementation** of the PRWM (Table 2).

**Table 2:** Outcomes, data collected to measure the outcome and its analysis.

Outcome	Measure	Data collection	Data analysis
Adaptation	Adaptation was measured as any changes made to PRWM during the implementation process.	Reflective notes from conversations, reflection meetings and educational meetings. Observations on multiprofessional patient discussions. Documents produced by participatory teams.	All adaptations were tracked throughout the implementation process and synthesized and classified using FRAME.
Acceptability	Perception among implementation stakeholders that PRWM is agreeable, palatable, or satisfactory.	Reflective notes from conversations, reflection meetings, educational meetings, and coaching sessions. Observations on multiprofessional patient discussions.	Deductive qualitative analysis guided by Proctors framework.
Adoption	The intention, initial decision, or action to try or employ PRWM among implementation stakeholders.	Reflective notes from conversations, reflection meetings, and coaching sessions. Observations on multiprofessional patient discussions.	Deductive qualitative analysis guided by Proctors framework.
		Logbook on participatory teams.	Descriptive analysis.
Feasibility	The extent to which implementation stakeholders perceived PRWM can be used or carried out within district teams, a hospice and nursing home setting.	Reflective notes from conversations, reflection meetings, educational meetings, and coaching sessions. Observations on multiprofessional patient discussions.	Deductive qualitative analysis guided by Proctors framework.
Fidelity	The degree to which PRWM was implemented by implementation stakeholders as it was intended by the research team.	Reflective notes from conversations, reflection meetings, educational meetings, and coaching sessions. Observations on multiprofessional patient discussions. Documents produced by participatory teams.	Deductive qualitative analysis comparing intended and actual implementation processes guided by Proctors framework.
Sustainability	The extent to which PRWM is maintained or institutionalized within a service setting’s ongoing, stable operations.	Reflective notes from conversations, reflection meetings, and closing conversation.	Deductive qualitative analysis guided by Proctors framework.
		Survey managers.	Descriptive analysis
Implementation processes shaping implementation outcomes	Contextual determinants	Reflective notes from conversations, educational meetings, coaching sessions, reflection meetings, and closing conversations; Observations on multiprofessional patient discussions.	Deductive qualitative analysis using CFIR.
	Implementation strategies		Deductive qualitative analysis using ERIC.
	Mechanisms of action		Deductive qualitative analysis using COM-B.
HCPs perceived impact of PRWM.	Healthcare professionals’ perceptions of the impact and added value of PRWM in practice.	Reflective notes from conversations, reflection meetings, educational meetings, coaching sessions, and closing conversations. Observations on multiprofessional patient discussions.	Thematic qualitative analysis.

The Framework for Reporting Adaptations and Modifications-Expanded (FRAME) was used as a guide to document the adaptation process [15]. FRAME is a comprehensive framework that enables documentation of adaptations made to an intervention, and it includes the following: (1) when and how in the implementation process the modification was made, (2) whether the modification was planned/proactive (i.e., an adaptation) or unplanned/reactive, (3) who determined that the modification should be made, (4) what is modified, (5) at what level of delivery the modification is made, (6) type or nature of context or content-level modifications, (7) the extent to which the modification is fidelity-consistent, and (8) the reasons for the modification, including (a) the intent or goal of the modification and (b) contextual factors that influenced the decision [15].

Implementation outcomes included **acceptability, adoption, feasibility, fidelity, and sustainability** of the PRWM within a nursing home, homecare and hospice setting and multiprofessional teams. The Implementation Outcomes Framework was used as a taxonomy of implementation outcomes to conceptualize and measure implementation [16].

In addition, **implementation processes** shaping implementation outcomes, such as contextual determinants, implementation strategies, and mechanisms of action, were examined. The Implementation Research Logic Model (IRLM) served as a practical framework to structure data analysis and synthesis [17]. The IRLM is a semi-structured, principle-guided tool designed to improve the specification, rigor, reproducibility, and testable causal pathways involved in implementation research projects [17]. The model provides a structured approach to examining and illustrating the relationships between determinants (barriers and facilitators), implementation strategies, mechanisms of action resulting from the strategies, and the implementation and clinical outcomes affected. The Consolidated Framework for Implementation Research (CFIR) was used as a determinant framework to identify factors that influence the intervention’s implementation. The CFIR includes five major domains each consisting of a number of constructs [18,19]: 1. Intervention: the features of the intervention that is implemented (ex. the relative advantage of implementing the intervention, adaptability and complexity of the intervention); 2. Outer setting: the features of the external context or environment (ex. relevant external policies and funding); 3. Inner setting: the features of the setting in which the innovation is implemented (ex. structural characteristics, relational connections, and culture); 4. Characteristics and roles of individuals involved (ex. Individual’s competence, knowledge, and skills to fulfill role); 5. Implementation process: the strategies or tactics used for the implementation of the intervention, such as planning, executing, reflecting, and evaluating. The Expert Recommendations for Implementing Change (ERIC) compilation of 73 strategies was used to determine which strategies would best address contextual barriers identified by CFIR [20] and the nine ERIC categories for further clustering: 1) evaluative and iterative strategies, 2) interactive assistance, 3) context tailoring, 4) stakeholder interrelationships, 5) training and education, 6) clinician support, 7) consumer engagement, 8) financial strategies, and 9) infrastructure changes [21]. Proctor’s guidelines for naming, defining and operationalizing implementation strategies were used in terms of seven dimensions: actor, the action, action targets, temporality, dose, implementation outcomes, and justification [22]. The COM-B system (Capability, Opportunity, Motivation–Behaviour), a framework for understanding behaviour, was used to identify mechanisms of action [23]. Within this framework, behaviour results from the interaction between capability, opportunity, and motivation. Capability refers to an individual’s psychological and physical capacity, including knowledge and skills; motivation refers to the processes that energize and direct behaviour; and opportunity refers to external factors that enable or prompt behaviour.

A secondary outcome focused on the perceived **impact of the PRWM on HCPs**, based on their own experiences with using the method. Patient-level outcomes were not included in this study.

### Data collection

Both qualitative and quantitative data were collected. Data were collected by two researchers (KK, EdG) and two coaches (GU, GH). The research team, contact persons and coaches reflected regularly on the process and the reflections made.

#### Qualitative data collection.

Data were collected during formal conversations, multiprofessional patient discussions, reflection meetings, educational meetings and closing conversations with contact persons and management (Table 3). These contacts were predominantly online. In addition, the notes made by the two coaches were also collected. Further, documents produced by participating teams related to the development and implementation of the PRWM were collected.

**Table 3:** Qualitative data collection.

Data collection during	Data	Prompts used	Dates of collection
Formal conversations (n = 36)	Reflective notes (KK)	Implications for adaptation and implementation; Implementation processes (barriers and facilitators, strategies, mechanisms).	Throughout the project
Educational meetings (n = 11 a 2hours)	Reflective notes (KK)	Objective; meeting structure; participation and interpersonal interactions; group dynamics; barriers and facilitators; implications for adaptations and implementation; reflection of teachers.	12.10.20 - 10.04.21
Coaching sessions (n = 96 a 30–60 minutes)	Reflective notes (GH, GU)	Objective coaching session; outcome and agreements; key observations.	Throughout the project
Multiprofessional patient discussions (n = 23 a 30–45 minutes)	Observations (non-participatory) (KK)	Goal; meeting structure; chairperson’s role; participation and interpersonal interactions; group dynamics; barriers and facilitators; notable observations that stood out; implications for adaptation and implementation; chairperson’s reflection.	Throughout the project
Reflection meetings (n = 47 a 2hours)	Reflective notes (KK, EdG);	Setting (online/offline, location, participants present, relevant contextual factors); meeting structure; participation of participants; group dynamics; reflection on execution of plans and team goals; supporting implementation tools; barriers and facilitators; other notable reflections that stood out; implications for adaptations and implementation.	08.12.20 - 27.01.22
Closing conversation with contact persons and management (n = 7)	Reflective notes (KK)	What went well and what could be improved during implementation; personal goal; sustainability within the participatory teams, at the site level, and organization-wide (plans, practical implementation, support needed from the research team, and support needed from colleagues and management); reflection on the implementation process as a whole.	13.01.22–25.04.22

#### Quantitative data collection.

At the start of the study, baseline characteristics of the organization, the participatory team, contact persons, and HCPs were collected. Throughout the implementation process, a logbook was used to monitor quantitative process indicators, such as the number of implementation cycles, coaching sessions, and multiprofessional patient discussions. At the end of the final action cycle, a brief survey was distributed to all team managers (n = 9) via SurveyMonkey. The eight-item survey assessed aspects of implementation and sustainability, including (1) awareness of the PAR process, (2) alignment of PRWM with organizational vision, (3) board and (4) management engagement, (5) future implementation plans, (6) facilitating and (7) hindering factors, and (8) sustainability strategies.

### Data analysis

Adaptations to PRWM were tracked throughout the implementation process. At the end of the study, all adaptations were synthesized and classified using the FRAME to describe the nature and rationale of the modifications [15].

The Implementation Research Logic Model (IRLM) served as a practical framework to structure data analysis and synthesis [17]. A stepwise approach was applied. First, data were analyzed using the five domains of the CFIR to get insight into barriers and facilitators [18,19]. All data was listed within the appropriate domain and underlying construct of the CFIR to identify elements that could be considered as facilitator or barrier for implementing PRWM in homecare, hospice and nursing home settings. Key findings were refined through iterative team discussions.

Second, CFIR-informed data were linked to implementation strategies, using the ERIC taxonomy of 73 strategies [20], and its effect on behaviour change of stakeholders was analyzed using COM-B [23]. Further, strategies were clustered into the nine ERIC categories [21] and specified following Proctor et al.’s seven dimensions [22].

Lastly, implementation outcomes, acceptability, adoption, feasibility, fidelity, and sustainability, were analyzed using Proctor’s framework [16]. The implementation outcomes served as predefined coding categories, and relevant data were assigned to the appropriate category and analyzed to assess the extent to which each outcome was achieved. Data related to HCPs’ perceived impact of PRWM were pooled across data sources and analyzed inductively using thematic analysis. Codes were generated from the data and subsequently grouped into categories and themes.

Finally, all analytical steps were integrated into the IRLM, capturing: (1) determinants (CFIR), (2) implementation strategies (ERIC), (3) mechanisms (COM-B), (4) implementation outcomes (Proctor), and (5) the intervention itself [17,24].

All steps in the analytical process were performed by two researchers (KK, EdG). Findings were discussed on regular basis, until consensus was reached on the interpretation of the findings. Data on the survey were exported from SurveyMonkey into Microsoft Office Excel version 2016 and analyzed using descriptive statistics.

### Reflexivity

The study was conducted by a multidisciplinary research team, all female and white, including an epidemiologist with qualitative research training (KK), an epidemiologist and senior qualitative researcher (EdG), a nurse practitioner in palliative care and palliative care consultant (GH), and a senior researcher who is an oncology nurse (ST). A broader project group advised the research team on patient and relative perspectives, HCPs perspectives, care practices, and research. A patient-and family advisory council further represented the perspectives of patients and relatives. The participating teams were not known beforehand to the research team. Throughout the study the participating teams had a changing composition of people, as well as that some contact persons and managers changed. Reflection on the process was done by the research team, contact persons and coaches. Most contact with HCPs, contact persons and managers was online.

### Ethical considerations

This study was conducted following the principles of the Declaration of Helsinki [25] and the General Data Protection Regulation [26]. The Utrecht Medical Research Ethics Committee classified this study as exempt from the Medical Research Involving Human Subjects Act (WAG/mb/20/005526). Participatory teams were recruited between 1 September 2019 and 12 October 2020. All teams received oral information and a letter with information about the content of the study as well as confidentiality of the data. Written and verbal informed consent was obtained from all participants. Verbal consent was obtained at the start of the study during the kick-off meeting, witnessed by the action group and documented in the study file, and was reaffirmed at each reflection meeting. Written informed consent was obtained for each sub-study. A contract was signed between the researchers and the organization, as well as a confidentiality agreement and hosting agreement from all researchers collecting data.

## Results

### Participatory teams

In total 17 nursing home teams and six home district teams were included in the study. Of these, 17 nursing home teams (13 psychogeriatric wards, three somatic wards, and one palliative unit) and three home district teams, including one working in a hospice, from seven organizations completed the study. Two district teams dropped out before the start of the study due to problems with organizing the study; one district team dropped out after three months due to high work pressure. The 20 remaining teams formed 12 participatory teams with 21 contact persons, most of whom were nurses (76%) (Table 4). At the start of the study, 262 HCPs were involved, with participatory teams ranging from 13 to 35 members, consisting mostly of nurse assistants (74%) and nurses (14%). Most HCPs had not received palliative care training from the organization and were unfamiliar with Decision making in the Palliative Phase and/or Signaling in the Palliative Phase. Most participatory teams (11 out of 12) completed three action cycle rounds. Across the 12 participatory teams, 197 reflective notes of conversations, educational meetings, coaching sessions, and reflection meetings as well as 23 observations on multiprofessional patient discussions were analyzed (Table 2).

**Table 4:** Characteristics of contact persons and HCPs in participatory teams.

	Contact persons N = 21	
	N	%
Nurse (level 4&6)	11	52
Nurse specialized in PC (master, level 7)	5	24
Nurse assistant (level 3)	4	19
Elderly care physician	1	5
	**HCPs** N = 262*	
	**N**	**%**
Nurse assistant (level 3)	99	38
Nurse assistant (level 1)	60	23
Nurse assistant (level 2)	35	13
Nurse (level 4)	28	11
Nurse (bachelor level 6)	7	3
Chaplain	6	2
Psychologist	5	2
Elderly care physician	4	2
Physiotherapist	4	2
Specialized nurse in PC (master, level7)	3	1
General practitioner	3	1
Occupational therapist	3	1
Speech therapist	2	1
Music therapist	2	1
Social worker	1	0.4

HCPs: healthcare professionals; PC: Palliative Care.

* At the start of the study.

### Adaptation

During the implementation process, several adaptations were made to the PRWM. In total, fourteen content modifications were identified in Palliative Reasoning (Table 5). The nature of these modifications included substituting (n = 2), tailoring/tweaking/refining (n = 10), and adding elements (n = 2). In addition, seven supporting materials were developed as implementation and scale-up activities to support consistent application and facilitate use in daily practice.

**Table 5:** Adaptations (n = 14) made to Palliative Reasoning coded according to FRAME.

Content modifications made to Palliative Reasoning during implementation							
Nature of content modification	What was modified	Were adaptations planned?	Who participated in the decision to modify?	For whom/ what is the modification made?	Relationship fidelity/core elements	Reasons for the modification	
						Intent/goal of the modification	Contextual factors that influenced the decision
**Substituting**							
Terminology (Step 3) (N = 2)	Within Step 3, “By whom” was reformulated as “Who will conduct the evaluation”, and “Time frame” was reformulated as “The next evaluation moment”.	Planned	Participatory teams and researchers	HCPs	Fidelity consistent	To improve clarity and feasibility by explicitly defining responsibility for and timing of the evaluation process.	/
**Tailoring/tweaking/refining**							
Increased emphasis on **assessment tools** (Step 1) (N = 3)	The use of assessment tools, which was already incorporated in the original Palliative Reasoning method as a general principle applicable to each step, was further integrated and made more explicit throughout the adapted version. In Step 1, symptom monitoring using the USD-4D was incorporated within symptom assessment. In addition, greater emphasis was placed on the use of assessment tools in Step 3. Within the evaluation of intervention effects, the items “How to measure the effect (assessment tool)” and “Use appropriate assessment tools” were added to support structured evaluation and monitoring of outcomes.	Planned	Participatory teams, researchers, coaches	HCPs	Fidelity consistent	To improve the feasibility and consistency of palliative reasoning by explicitly integrating assessment tools, supporting structured symptom monitoring (USD-4D), and providing HCPs with guidance for systematic assessment, monitoring, and evaluation.	Previous training and skills
Increased emphasis on **informal caregivers** (Step 1) (N = 2)	Although the original Palliative Reasoning method already emphasized alignment with patients and their informal caregivers throughout all steps (“Consult with the patient and informal caregivers”), the role of informal caregivers was made more explicit in Step 1. Informal caregivers were specifically included alongside patients when assessing the meaning of symptoms and identifying wishes and priorities.	Planned	Participatory teams, researchers, Coaches	Informal care providers HCPs	Fidelity consistent	To strengthen the visibility and voice of informal caregivers in the assessment process and prevent their perspectives, wishes, and priorities from being overlooked.	Previous training and skills Social climate (culture/climate)
Increased emphasis on **shared decision making** (Step 2 and 4) (N = 2)	Shared decision-making was given greater emphasis by explicitly incorporating it into the process of problem prioritization and proactive care planning. In Step 2, the item “Make a joint decision” was added to support shared decision-making between HCPs, patients, and, where appropriate, informal caregivers. In Step 4, shared decision-making was further reinforced by revising the instruction “Adapt the care plan as needed and constantly evaluate” to “Adapt the care plan as needed in consultation with the patient and constantly evaluate”.	Planned	Participatory teams, researchers, Coaches	Patients, informal care providers, and HCPs	Fidelity consistent	To address the need identified by the intellectual owners for greater attention to shared decision-making. During the implementation process, this need was further explored within participatory teams to determine how and where shared decision-making could be explicitly embedded within palliative reasoning, thereby increasing awareness and supporting consistent patient involvement in decision-making.	Previous training and skills Social climate (culture/climate)
Increased emphasis on **advance care planning** (Step, 2, 3, and 4) (N = 3)	Although advance care planning was already implicitly reflected in Step 2 through the prompt “Think proactively”, the initiation and continuation of advance care planning as an ongoing process were more explicitly emphasized and integrated throughout the decision-making process (Steps 2–4). Specifically, Step 2 was expanded to include “Initiate an advance care planning conversation”; Step 3 included “Follow-up of advance care planning”; and Step 4 included “Discuss advance care planning”.	Planned	Participatory teams, researchers, coaches	Patients, informal care providers, and HCPs	Fidelity consistent	To address the need identified by the intellectual owners for greater attention to advance care planning. During the implementation process, this need was further explored within participatory teams to determine how and where advance care planning could be explicitly embedded within palliative reasoning to increase awareness and facilitate the systematic integration of advance care planning by explicitly embedding its initiation, follow-up, and discussion throughout palliative reasoning.	Previous training and skills Social climate (culture/climate)
**Adding elements**							
Explicit attention to **adequate documentation and handover** (Step 3, at each step) (N = 2)	Greater emphasis was placed on documentation and handover throughout palliative reasoning. In Step 3, the item “Make agreements about the evaluation of the care plan and document them” was added. In addition, the instruction “Ensure adequate documentation and effective handover” was incorporated across all steps to promote continuity and communication of care.	Planned	Participatory teams, researchers, coaches	Patients, informal care providers, and HCPs	Fidelity consistent	To increase HCPs awareness of the importance of documentation and handover, thereby supporting continuity, coordination, and communication of care.	Previous training and skills Available resources (technology)

The adapted PRWM consists of three components:

1. Patient identification
2. Palliative Reasoning (updated version; Table 6)
3. Supporting materials

**Table 6:** Palliative reasoning before and after adaptation.

	Palliative Reasoning before adaptation	Palliative Reasoning after adaptation
Step 1	Assess situation in the light of a new symptom/concern or changed situation	Assess situation in the light of a new symptom/concern or changed situation
	Inventory:	Inventory:
	Medical history: - Diagnosis, comorbidities, treatment	Medical history: - Diagnosis, comorbidities, treatment
	Patient assessment across four dimensions: - Initial impression of the situation (who do I see when I enter the room?) - Physical, psychological, social, existential	Patient assessment across four dimensions: - Initial impression of the situation (who do I see when I enter the room?) - Physical, psychological, social, existential
	Estimated life expectancy	Estimated life expectancy
	Medication currently being used by the patient	Medication currently being used by the patient
	Symptom analysis - Anamnesis - Physical examination	Symptom analysis - Anamnesis - Physical examination **- Monitoring symptoms (USD-4D)**
	Meaning of the symptoms for the patient	Meaning of the symptoms for the patient and **informal caregivers**
	Wishes and priorities of the patient	Wishes and priorities of the patient and **informal caregivers**
Step 2	Summarize problems and formulate a proactive care plan	Summarize problems and formulate a proactive care plan
	Prioritize complaints and symptoms	Prioritize complaints and symptoms
	Formulate a working hypothesis - Nature of the symptom - Underlying cause of the symptom - Contributing factors	Formulate a working hypothesis - Nature of the symptom - Underlying cause of the symptom **-** Contributing factors
	Define the goal of care	**Define the goal of care**
		**Make a joint decision**
	Develop a care plan: - Treatment of the underlying cause - Symptomatic treatment - Non-pharmacological and pharmacological interventions - Supportive care	Develop a care plan: - Treatment of the underlying cause - Symptomatic treatment - Non-pharmacological and pharmacological interventions - Supportive care
	Think proactively	**Initiate an advance care planning conversation**
Step 3	Make agreements about the evaluation of the care plan	Make agreements about the evaluation of the care plan **and document them**
	Evaluate the effect of each intervention: - Agree on and plan: ◦ By whom ◦ Timeframe - Assess the effect on symptoms and concerns, experiences, functioning, well-being, values and wishes	Evaluate the effect of each intervention: - Agree on and plan: **◦ The next evaluation moment** **◦ Who will conduct the evaluation** ◦ **How to measure the effect (assessment tool)** - Assess the effect on symptoms and concerns, experiences, functioning, well-being, values and wishes **- Use appropriate assessment tools**
		**Follow-up of advance care planning**
Step 4.	Adapt the care plan as needed and constantly evaluate	Adapt the care plan as needed **in consultation with the patient** and constantly evaluate
	Determine the effect: - Effect adequate: ◦ Continue periodic evaluation → return to step 3 - Effect absent or limited: ◦ Adjust the care plan → return to step 2 ◦ Reassess the situation and revise the working hypothesis → return to step 1 ◦ Accept, communicate, and provide support	Determine the effect: - Effect adequate: ◦ Continue periodic evaluation → return to step 3 - Effect absent or limited: ◦ Adjust the care plan → return to step 2 ◦ Reassess the situation and revise the working hypothesis → return to step 1 ◦ Accept, communicate, and provide support
		**Discuss advance care planning**
At each step	Consult with the patient and informal caregivers.	Consult with the patient and informal caregivers.
	Consider the use of assessment tools.	Consider the use of assessment tools.
		**Ensure adequate documentation and effective handover.**
**Bold =** content modifications made.		

Several supporting materials were developed, including:

- guideline for HCPs including the following: a) specification of steps and sub-steps of Palliative Reasoning, b) detailed manual per step and sub-step for HCPs (detailed explanation, example questions, who is involved, what responsibilities per discipline), and c) additional information (guidelines, websites, films, books) (S1 Table);
- guideline for the chair of multiprofessional patient discussions;
- worksheet for HCPs to use before and during these meetings;
- Utrecht Symptom Diary – 4-dimensional manual for HCPs;
- informational materials for patients and their relatives, such as an introductory letter on Palliative Reasoning and Utrecht Symptom Diary – 4-dimensional;
- implementation guideline for organizations; and information standard, version 1.0 including an implementation guide to achieve integrated reporting and registration.

### Implementation

**Fig 3:**
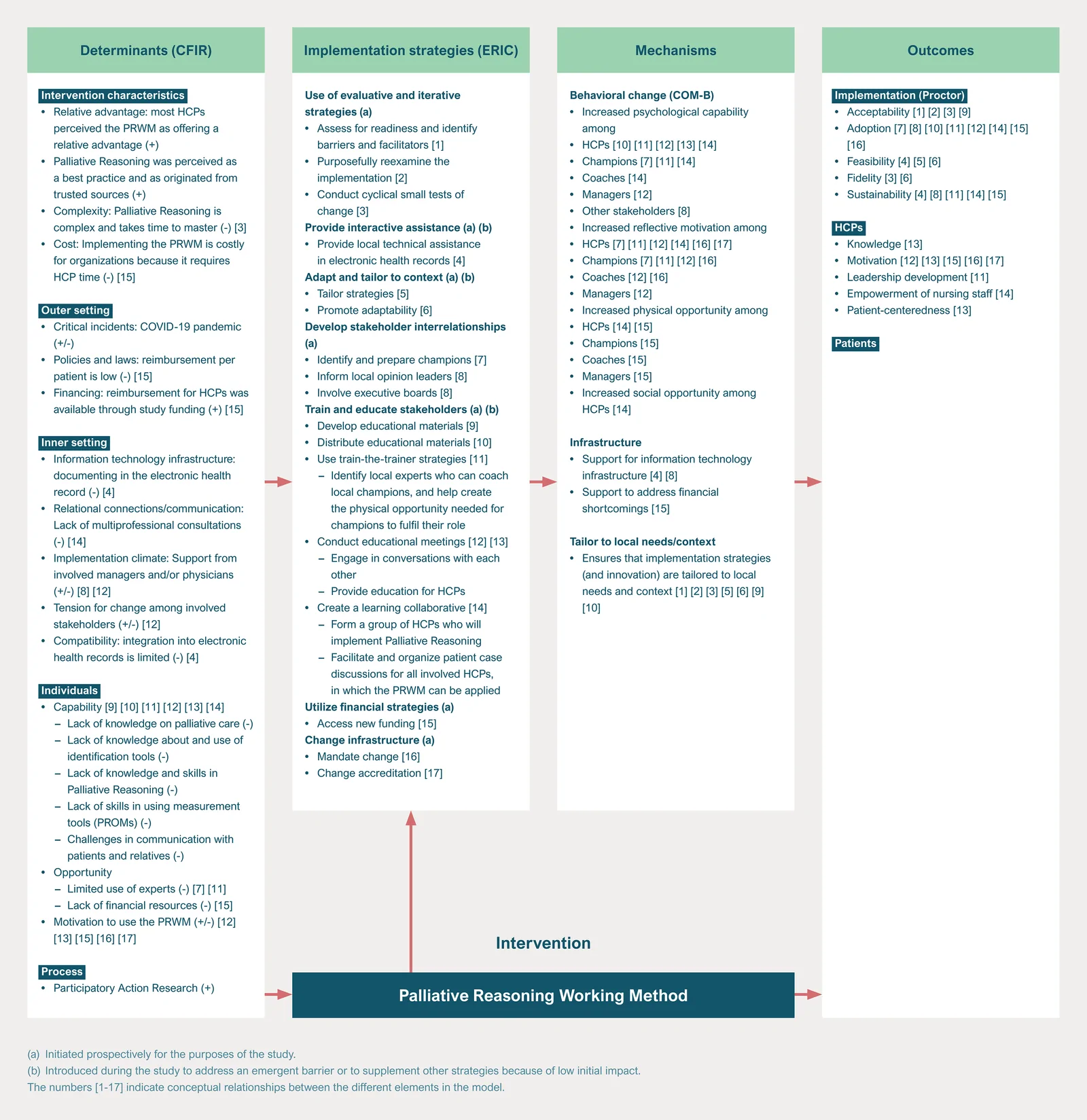
Implementation Research Logic Model (IRLM) of Palliative Reasoning Working Method.

A logic model was developed to provide a graphic depiction presenting the shared relationships among various elements of PRWM [17] (Fig 3). This model includes determinants of implementation, implementation strategies, mechanisms of implementation and outcomes.

#### Determinants of implementation.

The implementation of the PRWM was found to be influenced by determinants across the five CFIR domains [18,19]:

1. At the intervention level, HCPs reported positive experiences with the PRWM, recognizing its clear added value and practical usefulness in daily care. Yet, it was also considered complex and time-consuming to learn and integrate into daily practice.
2. Outer setting factors: the Covid-19 epidemic and financing structures had a negative impact on implementation.
3. Within the inner setting, the availability of appropriate information technology infrastructure (documentation in electronic health records), existing relational networks and communication, and the degree of leadership engagement and support were particularly influential.
4. At the individual level, capability, including knowledge, skills, and attitudes regarding palliative care and Palliative Reasoning, as well as motivation and perceived opportunity to apply PRWM in practice, affected HCPs ability to engage with the intervention.
5. Regarding the process, a structured implementation process (PAR), with attention to participation and communication, was essential to support adoption of the PRWM.

#### Implementation strategies and mechanisms.

A total of 17 of the 73 strategies according to the ERIC taxonomy [20], were found to support the implementation of PRWM. They were grouped into seven of the nine ERIC categories (S2 Table): ‘train and educate stakeholders’ (n = 5 strategies), ‘use evaluative and iterative strategies’ (n = 3), ‘develop stakeholder interrelationships’ (n = 3), ‘adapt and tailor to context’ (n = 2), ‘change infrastructure’ (n = 2), and ‘provide interactive assistance’ and ‘utilize financial strategies’ (n = 1 each).

Three mechanisms were found to be important to implement PRWM:

1 Behavioral change

Implementation of PRWM primarily relied on behavioral change mechanisms (COM-B), particularly to enhance uptake and use in daily practice. A key strategy was to ‘identify and prepare champions’, often nurse assistants, registered nurses, or palliative care specialists. Champions significantly shaped implementation outcomes, driven by their capability and motivation. Their informal influence on team attitudes, psychological capability (e.g., understanding of palliative care and Palliative Reasoning), leadership skills, and both automatic and reflective motivation were critical factors. To ensure continuity, appointing two to three champions per team was recommended. Most behavioral change strategies focused on education, aiming to enhance psychological capability and reflective motivation among HCPs, champions, and managers. A train-the-trainer approach, led by skilled palliative care nurses (GH and GU), supported champions in developing leadership competencies. Additional strategies included developing and distributing educational materials, conducting training sessions, and establishing learning collaboratives (multiprofessional patient discussions); key to addressing the knowledge gap identified as a major barrier.

Learning collaboratives primarily engaged nursing staff, with limited participation from other disciplines, largely due to lack of time (physical opportunity) and unclear understanding of the change process and professional roles. Opinion leaders and managers strongly influenced implementation success. A lack of managerial commitment negatively impacted HCPs’ and champions’ reflective motivation. Strategies such as developing stakeholder interrelationships, providing information, and maintaining ongoing dialogue with reluctant stakeholders, HCPs, and managers helped to overcome these barriers.

2 Change of context

Other strategies operated through mechanisms of action aimed at changing the context by tailoring and refining both the PRWM and implementation processes to local needs and possibilities. In this study, key strategies included assessing barriers and facilitators for each team, regularly reviewing implementation progress, applying iterative adjustments in small cycles, and adapting all educational materials accordingly.

3 Changes in infrastructure

Lastly, strategies targeting organizational infrastructure were employed, including local technical assistance for integrating PRWM into electronic health records and securing additional funding to address financial constraints. Both were identified as preconditions for future implementation and should be considered essential to the implementation process. In this study, only three out of seven organizations (eight participatory teams) integrated elements of PRWM into their health records, which facilitated its practical application, such as record keeping according to the Palliatieve Reasoning methodology and incorporating PROMS into the system.

#### Implementation outcomes.

**Acceptability:** Overall, most HCPs perceived PRWM as appropriate and acceptable, primarily due to clear advantages observed in daily practice. They perceived benefits: not only improvement of quality-of-care but also a shift of professional roles and team dynamics.

The advantage of PRWM was perceived within several distinct areas:

a Identification of patients with palliative care needs

PRWM increased awareness of patient’s palliative care needs. The use of the Surprise Question enabled earlier recognition and timely initiation of appropriate care.

*“Through Palliative Reasoning, there was awareness of the palliative phase, making it possible to recognize and discuss key points.*” *(nurse, district team)*

b Enhanced person-centered care

The person-centered and four-dimensional approach allowed HCPs to better understand individual wishes and needs, placing patients’ priorities at the center of care.

*“We are now prioritizing things that are important to residents. Residents come first.” (nurse, nursing home)*

*“The residents are truly at the center now.” (nurse assistant, nursing home)*

*“We are accepting things that we would never have done otherwise. In our profession, we always want to have everything under control. But we have realized that the resident is not the problem but ourselves, how we handle situations. Should Mrs. not eat a cookie now because we find it stressful?!’ (nurse, nursing home)*

c Emancipation of nursing staff

PRWM strengthened the professional identity and confidence of the nursing staff, particularly in multiprofessional discussions. They felt more empowered to voice observations and contribute to decision-making; highlighting their critical role in palliative care.

d Improved collaboration

Many HCPs noted that they experienced a sense of unity and shared purpose for the first time. They were able to get to know each other, to acknowledge and recognize personal insecurities or a lack of knowledge and/or competences, and to acknowledge each other’s expertise.

*“There is more clarity within the team; we are all heading in the same direction.” (nurse, district team)*

*“We’ve been thrown together. You can’t do it alone. You need each other to really see and understand the resident.” (psychologist, nursing home)*

*“I’m so happy we’re participating in this project. For the first time, I truly feel like we are one team. Before, it felt more like separate little teams.” (spiritual counselor, nursing home)*

Despite these perceived advantages, PRWM was not universally experienced as suitable or acceptable. Several factors limited acceptance, particularly in the initial phases.

First, PRWM was perceived as a complex and demanding intervention. Many participants had limited prior knowledge of palliative care, and learning to apply PRWM required time, extensive explanation, and ongoing support. Assessing life expectancy and identifying palliative care needs using the Surprise Question was unfamiliar to most HCPs, and articulating these considerations proved challenging for many. The use of PROMs such as the Utrecht Symptom Diary – 4 dimensional (USD-4D) was experienced as time-consuming and emotionally demanding; some nurses struggled to initiate sensitive conversations and worried about burdening patients.

Second, convincing other professional groups, such as physicians’, spiritual carers, psychologists, and occupational therapists, of their role in palliative care proved difficult. They initially questioned the relevance of PRWM to their practice. Several physicians perceived it primarily as additional work, contributing to lack of engagement. However, over the course of the implementation, most other professional groups became involved, even if not always directly within the participatory team but rather on the sidelines. For instance, nurses and nurse assistants consulted other professionals whenever clinical decisions or care planning required their expertise. This indirect involvement allowed a broader engagement with the PRWM.

Finally, for some HCPs, PRWM continued, even toward the end of implementation, to feel like an extra task rather than an integral part of palliative care. A subset struggled to recognize that PRWM formalizes practices they already carry out, and to see it as already embedded within routine care delivery.

Overall, these barriers account for variations in acceptance: some incorporated PRWM into practice, while others were reluctant, related to workload, limited knowledge of palliative care, difficulties in using PROMs, and uncertainty about their roles within palliative care. Over time, as familiarity increased and positive outcomes were observed, acceptance became more widespread, though not universal.

**Adoption:** Adoption was high: of the 15 teams that intended to implement the method, 12 completed the full implementation process. The initiative leaders, such as local managers and specialized nurses in palliative care, and the champions demonstrated notable enthusiasm and determination, actively promoting PRWM within their organizations and holding numerous discussions with staff and management to ensure adoption. However, not all individual HCPs shared this enthusiasm or intention to try; many physicians were initially reluctant to participate actively in the implementation, and other non-nursing staff also engaged only minimally at the start, reflecting variations in acceptability across professional groups.

Notably, adoption occurred despite the challenging context of the COVID-19 pandemic, which increased workloads and caused staffing shortages. These pressures, while significant, did not prevent teams from initiating and sustaining the use of PRWM.

**Feasibility:** PRWM was perceived as feasible within nursing homes, district teams, and hospices. The working method is compatible with established workflows. Participatory teams used PRWM across various everyday clinical structures: conducting anamnesis for new patients; within multiprofessional patient discussions as a guide for preparation, execution and documentation; as an aid for structuring thought and discussion during critical decision-making moments; and facilitating daily or periodic communication with patients and their relatives regarding relevant care decisions. However, it is important to note that documentation in the electronic health record was perceived as a barrier in the use of PRWM, as the method was not integrated into electronic health records in most teams. Further, multiprofessional meetings/patient discussions on a regular basis to adopt PRWM improved feasibility. In nursing home teams, multiprofessional meetings or patient discussions needed to be established, as existing meetings typically occurred only every six months and did not involve all relevant team members. In district teams, space had to be created within team meetings to accommodate patient discussions.

Furthermore, PRWM also demonstrated to be trialable. One team discontinued the use of PRWM after three months without causing any lasting harm or disruption to care practice. This outcome indicates that PRWM can be safely tested in a local context, such as within a single team or department, before broader implementation is considered.

**Fidelity:** PRWM was largely implemented as intended by the research team. However, there were some deviations from the original implementation plans due to COVID-19: most meetings were held digitally rather than in person, both within the participatory teams and the action group; and one of the twelve participatory teams completed implementation in two action cycles instead of three. In addition, the content of each action cycle and the educational materials varied between teams. This demonstrates that the implementation of PRWM is flexible and can be adapted to local contexts and needs.

**Sustainability:** All participatory teams were eager to continue using and integrate PRWM into workflows and organizational practices after the study period. Managers (n = 9) made plans to implement PRWM across other wards (67%), locations (22%), or organization-wide (33%). The top three sustainability strategies identified were: integrating PRWM into broader organizational palliative care policies (77%), allocating time for HCPs to use PRWM (56%), and supporting enthusiastic HCPs in further implementation (56%). Although several strategies were employed during the study to support sustainability (e.g., technical assistance, involving opinion leaders, and securing funding), these were insufficient. From the managerial perspective, barriers identified were employee turnover (77%), insufficient financial resources (44%), and limited integration of PRWM into electronic health records (22%).

**Impact on HCPs:** HCPs involved stated that the implementation of the PRWM enhanced their understanding of palliative care principles, including identification of patients with palliative care needs, multidimensional symptom management, the use of PROMs, decision-making, and advance care planning. Furthermore, they felt more capable of providing patient-centered care.

Additionally, there was an increase in motivation among nurses and nurse assistants, along with the empowerment of nursing staff, as reported by themselves. Champions also demonstrated enhanced leadership skills. These developments also contributed to increased job satisfaction among the participating professionals.

## Discussion

This article describes the iterative adaptation and implementation of PRWM, an integrated intervention for multiprofessional teams in primary care, hospices and nursing homes. The PRWM consist of three elements: 1. Patient identification, 2. Palliative Reasoning (updated version), and 3. Supporting materials. In this study, adaptation and implementation of PRWM are described, including determinants, implementation strategies, mechanisms and outcomes of implementation.

### Palliative Reasoning Working Method

The PRWM was developed for use by multiprofessional teams and represents a palliative care approach that aligns with the core elements of the Dutch Quality Framework for Palliative Care [2]. The findings of this study show that the PRWM was perceived as both acceptable and feasible by most participating HCPs. Although some experienced the method as complex and time-consuming, its perceived benefits were substantial. The working method was considered compatible with existing workflows, and the perspectives of multiprofessional teams across primary care, hospices, and nursing home settings were found to align closely with the principles of the PRWM. Adoption of the method was generally high. Incorporating a palliative care approach into primary and nursing home care is essential, as all HCPs encounter individuals with palliative care needs and must be able to provide generalist palliative care. The PRWM’s supporting materials and the implementation strategies identified in this study can assist organizations and teams in applying a palliative care approach, thereby facilitating its ongoing integration into routine practice and organizational processes.

According to the HCPs involved, the PRWM supported them in working collaboratively within multiprofessional teams to systematically assess patients in a holistic manner and to formulate care plans based on the wishes, values, and needs of patients and their relatives. Furthermore, they reported that the PRWM facilitated earlier recognition of palliative care needs and strengthened person-centered care within the team. Several studies indicate that patients in the palliative phase have a strong need for a person-centered approach, explicitly expressing the desire to be seen and recognized as a person and to receive care that addresses their individual needs [5,27–32]. This highlights the importance of engaging in meaningful conversations with patients and their relatives, and of getting to know the patient across all dimensions of their experience. Core elements of the PRWM, such as the Surprise Question for early identification and the USD-4D to structure assessment and monitoring, were perceived as particularly valuable in providing person-centered care by enhancing symptom management, communication, and advance care planning. Considering that 23.6% of patients in the palliative phase in the Netherlands receive potentially non-beneficial treatments in the last month of life [1], the explicit integration of advance care planning, shared decision-making, and timely identification of palliative needs within the PRWM may support care that is better aligned with patients’ goals and preferences. Future research should evaluate whether this translates into reductions in non-beneficial care. Overall, these outcomes align with existing evidence emphasizing the value of structured needs assessment, advance care planning, and interprofessional collaboration in improving decision-making, symptom management, and person-centered palliative care [33–41].

### Implementation process

This study demonstrated that the PRWM can be implemented in district teams, hospices, and nursing homes. However, integrating the PRWM into routine workflows requires considerable organizational effort and commitment from HCPs. A substantial behavioral and mindset shift is needed, including consistent use of the Surprise Question and PROMs, structured interprofessional collaboration, and clear role enactment within the team; practices that are not yet embedded in everyday work routines. Limited pre-existing palliative care knowledge further contributed to the perception that applying PRWM was complex and added to workload, particularly in the early stages of adoption. As a result, acceptability must be actively nurtured within organizations, since the benefits of the PRWM become evident gradually rather than immediately. These findings are consistent with previous implementation studies in palliative care, which have shown that the integration of PROMs, advance care planning, and interprofessional collaboration into routine practice requires substantial behavioral, cultural, and organizational change, as well as ongoing training and leadership support [42–46]. While implementation of the PRWM was perceived to require substantial behavioral and organizational change, our findings also identified important facilitators. In line with previous research, motivated champions, supportive managers and physicians, integration into electronic health records, and adequate structural funding were considered essential for successful implementation and long-term sustainability [43,47].

These findings highlight that successful implementation depends not only on the content of the intervention, but also on its alignment with the organizational context and the level of support provided to teams. Although the integration of the PRWM requires effort from both organizations and HCPs, this investment ultimately pays off in care that is not only of higher quality but also more efficient, ultimately saving time and resources.

#### Reflection on implementation strategies and their mechanisms.

Most implementation strategies that were found to support the implementation of PRWM focused on education and training, with behavioral change serving as the primary underlying mechanism of action. These strategies primarily influenced capability, through training, education, and coaching, and supported motivation by fostering reflection, coaching and team support. Opportunity, however, proved more challenging to influence, as it is largely determined by organizational factors such as available staff hours, financial resources and integration of PRWM into electronic health records. Given that opportunity strongly impacts both capability and motivation [23], these findings underscore the need to address structural and system-level conditions to optimize behavioral change and support sustainable implementation of the PRWM. A scoping review on how palliative care interventions are implemented in nursing homes and other long-term care facilities indicates that successful implementation depends on four key strategies: facilitation, education/training, internal engagement, and external collaboration. The study demonstrates that involving internal staff, providing flexible and context-specific training, and securing management support are crucial for achieving sustainable change, which is in line with our study [48]. In our study, facilitation was provided by champions, mostly nurses and nurse assistants, who took on the role of supporting the implementation and motivating their colleagues. Internal engagement involved active participation of HCPs and managers in the implementation process, which fostered a sense of ownership and commitment to the implementation.

#### Role of the nurse.

An essential finding of this study was the central role that nurses and nurse assistants played in the implementation of the PRWM. At the start of the process, many demonstrated hesitation and uncertainty regarding their role and position within the team, particularly nurse assistants. Over time, however, this changed markedly. As implementation progressed, they increasingly assumed responsibility, recognized their value, and became more aware of their distinctive contribution to palliative care. Their involvement was especially visible in early identification and signaling of changes in patients’ conditions. Due to their close and continuous contact with patients and relatives, they were able to observe subtle changes, recognize needs more rapidly, and build trusting relationships, often without realizing the extent of their existing expertise.

Within the teams, contact persons, predominantly nurses and nurse assistants, were appointed as champions. Throughout the project, they grew into this champion role, developing leadership skills and actively promoting the PRWM within their teams. Their proximity to patients and other HCPs, combined with their practical knowledge of the care context, positioned them as key drivers of adoption and integration of the PRWM. Through extensive training and coaching, including a train-the-trainer approach led by experienced palliative care nurses, they reported feeling more empowered in their professional roles and more confident in interprofessional decision-making, including collaboration with physicians. This empowerment led to greater professional emancipation, increasing nurses’ influence within the team and strengthening recognition of their expertise. These findings align with recent research highlighting the importance of nursing involvement and leadership in improving palliative care processes and outcomes [49–52].

### Strengths and limitations

A key strength of this study lies in the PAR approach, which enabled a cyclical process in which research, action, and evaluation were continuously interlinked. Intended users were actively and consistently involved, ensuring that PRWM was adapted in practice and tailored to local contexts. While a structured framework was provided, participatory teams and contact persons determined what was needed to apply PRWM in their own setting, reflecting a combined bottom-up and top-down approach.

Another strength is the diversity and representativeness of the settings. The study included a broad range of teams from both primary care (district teams), nursing homes, including somatic wards, psychogeriatric wards, palliative units, and hospices. This enhanced the generalizability of findings.

Data triangulation also contributed to the study’s robustness, with multiple data sources and knowledge-building processes. Continuous reflection was integral to the methodology, with regular reflective sessions within the action group, which included the research team, coaches, contact persons, and HCPs.

Although nurses and nurse assistants formed the core of the participatory teams, most physicians were only indirectly involved and reluctant to adopt PWRM. As a result, the findings primarily reflect the perspectives and experiences of nursing staff. However, this pattern is consistent with current practice in many primary care and nursing home settings, where physicians are not routinely involved in day-to-day palliative care team meetings and nurses often take a coordinating role.

The COVID-19 pandemic posed a considerable challenge. National lockdowns halted all study activities from March 2020 to October 2020. When activities resumed, all processes had to be conducted digitally. This shift impacted the ability to observe teams and maintain close contact, placing even greater emphasis on the role of contact persons and their capacity to engage teams. COVID-19 also increased workload and staff absences, but paradoxically, the crisis also highlighted the relevance of PRWM. Teams reported that they had never worked so structurally and collaboratively, with an increased focus on palliative care.

Conducting action research in a scientific context is both a strength and a challenge. Although the approach is rigorous and systematic, it places high demands on both researchers and participants. PAR requires structured working and the continual development of effective strategies. To ensure a comprehensive evaluation of the adaptation and implementation of PRWM, a stepwise analytical approach was used, culminating in the development of a logic model.

### Implications for practice

The PRWM offers multiprofessional teams a structured approach to strengthen the delivery of appropriate palliative care. To promote and embed a structural approach to palliative care within an organization, several strategies were identified to be essential. Implementation of a palliative care approach requires ongoing education to enhance knowledge, structured interprofessional collaboration in form of regular multiprofessional team meetings, and the appointment of well-prepared (nurse-led) champions supported by engaged management and physicians. In addition, sufficient resources, technical assistance to integrate the approach into electronic health records, and supportive organizational policies are essential to embed it into routine practice. Finally, ongoing reflection, iterative adaptation, and tailoring to the local context are crucial for achieving sustainable implementation.

Nurses play a pivotal role in coordinating palliative care processes and should be empowered by both organizational leadership and physicians to fulfil this role confidently. Embedding Palliative Reasoning in professional training curricula and organization-wide palliative care frameworks may further promote a shared and systematic approach across care settings. Future research should examine long-term patient outcomes, explore optimal strategies for interprofessional collaboration within the PRWM, and investigate integration within electronic health record systems to support scalability and sustainability.

## Conclusion

PRWM offers a promising approach to embed Palliative Reasoning into routine generalist palliative care in primary care, hospice and nursing home settings. It strengthens proactive, multidimensional and person-centered care, particularly by empowering nursing staff and fostering collaborative practice. Successful implementation requires strategies focused on behavioral change, supportive infrastructure, and adaptability to local needs. Sustainable implementation depends on adequate funding and information technology infrastructure.

## Supporting information

S1 Table Palliative Reasoning Working Method guideline for HCPs. (DOCX)

S2 Table Specification of implementation strategies (Proctors 7 Domains of an Implementation Strategy). (DOCX)
