# Supplementary material for: Implementing palliative reasoning in multiprofessional palliative care: A participatory action research in the Netherlands
Source: PLoS One. 2026 Aug 28;21(8):e0354089. doi: 10.1371/journal.pone.0354089 (PMC13524244; doi:10.1371/journal.pone.0354089)
Supplement: Supplementary file 1 [file pone.0354089.s001.docx]

| **Palliative Reasoning Working Method** | | | |
| --- | --- | --- | --- |
| *In this manual, the term “patient” is used for reasons of readability. Depending on the care setting, the term may also refer to a client or resident.* | | | |
| **What is Palliative Reasoning?** | | | **Additional information** |
| People receiving care at home or in nursing homes often have a limited life expectancy due to an incurable illness or increasing frailty. This includes, for example, people living with cancer, dementia, multiple sclerosis (MS), chronic obstructive pulmonary disease (COPD), heart failure, or Parkinson’s disease. The care provided to these individuals is referred to as palliative care, which aims to optimize quality of life. The palliative phase varies for each patient. For some, it may last a few weeks, while for others, it may extend over months or even years. Palliative care addresses not only physical symptoms, but also psychological distress, social concerns, and spiritual needs. Supporting the patient's loved ones is also an important part of this care. To effectively identify care needs across these dimensions and to provide appropriate support, a multidisciplinary team is essential. Healthcare professionals from various disciplines work together with the patient and their loved ones toward shared goals.  Palliative Reasoning is a method that supports healthcare professionals in improving the quality of palliative care, particularly regarding symptom management. It offers a structured way of thinking and acting, starting with identifying the palliative phase, and consists of four steps: identifying symptoms, wishes and needs; analyzing symptoms; developing a care plan; and evaluating and adjusting the plan as needed.  This methodical approach not only helps to provide personalized, appropriate care, but also strengthens communication and collaboration within the team. Palliative Reasoning is applied when a patient has been identified as being in the palliative phase, when a new symptom arises, or when the patient's condition changes. | | | WEBSITE: General principles of palliative care (Pallialine, 2017) <https://palliaweb.nl/richtlijnen-palliatieve-zorg/richtlijn/algemene-principes-palliatieve-zorg>  WEBSITE/BOOK: Netherlands Quality Framework for Palliative Care (IKNL/Palliactief, 2017) <https://palliaweb.nl/richtlijnen-palliatieve-zorg/richtlijn/kwaliteitskader-palliatieve-zorg-nederland> |
| **Step** | **Specification** | **Manual for health care professionals** | **Additional information** |
| **Identification of**  **patients with palliative care needs** | Surprise Question: “Would I be surprised if this patient was to die in the next year?” | A tool to support the identification of patients with palliative care needs is the use of the Surprise Question.  If you answer this question with “no,” the palliative phase begins. Conversations about palliative care can be initiated, and you can assess whether the patient requires a different approach.  It is important to involve the patient and their loved ones in this process and to talk with them about your observations and what the patient considers important now and in the future. Are they already expressing any concerns or wishes themselves?  Advance Care Planning  Advance care planning is a continuous and dynamic process of conversations about life goals and choices, and what care aligns with those goals—both now and in the future (Netherlands Quality Framework for Palliative Care, 2017). It is a process that healthcare professionals go through repeatedly.  (It is a reflective process that helps you consider the patient’s current situation and incorporate that into the care plan going forward.) | FILM: Identification of the palliative phase (PZNL, 2019)  <https://www.youtube.com/watch?v=FYu9iz_dCeY&feature=emb_title> |
|  |  |  | FIGURE: Spectrum of Palliative Care [Vissers, 2010] |
|  |  |  | WEBSITE: Talking about the end of life (KNMG, 2022)  <https://www.knmg.nl/advies-richtlijnen/knmg-publicaties/tijdig-praten-over-het-levenseinde.htm>  **Advance Care Planning**  WEBSITE: Thematic Page on Proactive Care Planning (Palliaweb, 2021) <https://palliaweb.nl/zorgpraktijk/proactieve-zorgplanning> |
| **SIGNALING** | | | |
| **STEP 1 – Assess situation in the light of a new symptom/concern or changed situation** |  | When you have identified a patient, notice a new symptom or concern, or when the patient’s situation changes, you start using the Palliative Reasoning Working Method. In STEP 1, you map out the patient’s individual situation; this information is then used during the multidisciplinary meetings. Based on this information, you jointly develop a care plan.  As preparation, consult with other disciplines if they cannot attend the meeting, to obtain the most complete picture possible. |  |
| 1) Medical history | Diagnosis, current and  previous therapy and  comorbidity | The medical history primarily falls within the domain of the physician.  Assess whether the symptoms/concerns are known or new.  Check if the patients knows and understands his/her situation. |  |
| 2) Multidimensional  assessment of the  patients current status | Use of a personal  patient profile | Who do I see when I enter the room?  Give a literal description of what you observe: think about whether the patient is in bed, dressed, in pajamas, engaged in contact, withdrawn, being cared for, etc.  Try to remain objective and avoid drawing conclusions.  Describe the current situation, including all relevant dimensions.  Life story:  If not done yet, this step can include gathering the patient’s life story. |  |
|  | Physical | - What is the patient still able to do? - What can the patient no longer do? When did the limitation arise, and does the patient experience discomfort or distress because of it? - Describe the physical condition. |  |
|  | Psychological | - Current cognitive status (intact or impaired, and in what way impaired; consciousness, understanding, attention, memory, knowledge and insight into the situation, need for control and autonomy) - Current emotional status (worries, anxiety, sadness, contentment, mood) |  |
|  | Social | - What roles does/did the patient have in daily life? What do these roles mean to the patient? Has the patient accepted the changes in these roles? Do they struggle with them? - Does the patient still provide care for someone else? - Describe the patient’s environment (family, neighbors, acquaintances, friends). Who are important? - Support for relatives: are there any issues or concerns affecting the patient’s loved ones? |  |
|  | Existential | - Patient’s beliefs (e.g., religious faith) and their significance - Meaning and purpose: what gives the patient strength? - Unfinished business: Are there any problems or past situations that cause distress because they remain unresolved or unspoken but that the patient would like to discuss? |  |
| 3) Estimated life  expectancy | Sets the scope for  interventions and the  related continuity of  care | It is important to continuously assess the patient’s life expectancy. This assessment helps to evaluate the relevance of (future) care interventions and guides the care plan.  Who assesses the life expectancy?  Depending on the situation, it is the healthcare professionals or the team who make this assessment. When using the Palliative Reasoning Working Method during a meeting, this can be discussed collectively.  The patient and their relatives may also indicate themselves that the patient has a limited time to live; this can be shared during the meeting and documented in the medical record (do not routinely ask the patient).  Engaging in conversation with the patient and relatives  Whenever possible, discuss the life expectancy and any changes in it with the patient and their relatives, so they are involved in the process and have time to reflect on their wishes, values, needs, and the care they desire. Pay attention to what the patient and their relatives need during this process. |  |
| 4) Medication currently (actually) being used | Current use of  Medication and  changes in medication | Medication mainly falls within the domain of the physician. |  |
| 5) Symptom analysis | Information from anamnesis, physical examination, and use of measurement tools | Anamnesis and physical examination primarily fall within the physician’s domain.  During the analysis, all information is combined: what the patient experiences alongside the anamnesis and physical examination findings (from the physician). Describe here the analysis of symptoms across all four dimensions that have been identified. Try to specify each symptom as precisely as possible (e.g., pain → which type of pain).  Use of measurement instruments (Pallialine, 2017):  Measurement instruments are standardized questionnaires designed to analyze symptoms in the most reliable way. These are usually completed by the patient, but sometimes by relatives or healthcare professionals.  Various instruments are available, which can be used for:   - Screening: identifying what the patient is experiencing (e.g., the Lastmeter). - Monitoring: tracking symptom progression over time and treatment effects (e.g., the Utrecht Symptom Diary - 4 dimensional, USD-4D). - In-depth assessment: standardized measurement with specific tools indicating the likelihood of a treatable diagnosis (e.g., depression or delirium). - Diagnosis: establishing a diagnosis based on objectively testable criteria (e.g., depression according to DSM V criteria).   If a measurement instrument has been used, the results can be presented here. Consider the following questions:   - What are the main conclusions? - Can the patient accurately report symptoms, or was a relative helpful in this? - Which symptoms stand out in the USD-4D? - Are there changes visible over time? - Is there a correlation between different complaints/symptoms? - Were other measurement tools used? What conclusions can be drawn from their data? | Measurement instruments  - WEBSITE: Symptom analysis (Pallialine, 2017) <https://palliaweb.nl/richtlijnen-palliatieve-zorg/richtlijn/algemene-principes-palliatieve-zorg/symptoomanalyse>    - BOOK: Measurement instruments in palliative care (IKNL, 2018) <https://iknl.nl/getmedia/61fe7295-2c96-4472-a647-d867ea760780/palliatieve-zorg-in-beeld-meetinstrumenten.pdf> |
| 6) Meaning of the symptoms | Gain insight into the meaning of the symptoms for the patient and their relatives | What do the experienced symptoms mean for the patient and their relatives? |  |
| 7) Values, wishes and  needs | Gain insight into the values, wishes and needs of the patient and relatives | This is not just about the symptoms, but a broader perspective:   - What are the patient’s priorities and trade-offs? What causes them the most burden, and at what cost? - Which symptoms/concerns trouble the patient the most? - What do the patient and their relatives want to prioritize? - What are their wishes and needs? - Besides specific symptoms, other aspects can be important as well, such as seeing certain people one last time or arranging practical matters. - It is also important to recognize that sometimes patients and their relatives may struggle with these questions and may be unsure of what they want or need. |  |
|  |  | Is there a discrepancy in priorities between the patient and their family? If so, discuss this with the team, the patient, and their relatives. |  |
| **DECISION-MAKING** | | | |
| **STEP 2 – Summarize problems and formulate a proactive care plan**  **STEP 3 – Make agreements about evaluation and record them** |  | Based on the information gathered above, you will now jointly discuss which symptoms and concerns are the most important to address, what the possible causes of these symptoms and concerns might be (working hypothesis), and you will make agreements about the care plan and evaluation. This is truly something you do together as a team. |  |
| 1) Prioritizing symptoms | Map the priorities of  the patient, relatives  and caregivers | - Which symptoms/concerns are the most important to address? - What symptoms/concerns cause the patient the most distress, and what are their priorities? - Select one or two symptoms/concerns to focus on—those where you can truly make a difference for the patient. - The challenge here is to choose. - What do the patient and their relatives consider important, and what do you observe as a professional? |  |
| 2) Formulate a  working hypothesis | Describe the nature of the symptom, including any possible underlying causes or influencing factors | A working hypothesis includes a symptom along with its (possible) cause(s) and any influencing factors. The care plan is based on this working hypothesis.  Assumption about the origin of the symptom: what seems most likely?   - Consider whether additional information is needed (e.g. internal/external consultation, use of assessment tools, or referring to palliative care guidelines). - Determining possible causes is something in which other disciplines (such as physicians) will collaborate.   Nature of the symptom: When and where does it occur? How long has it been present? How much distress does it cause? Are there connections with other symptoms?  Caused by: The most plausible cause(s) of the symptom.  Influencing factors: Factors that worsen or relieve the symptom. Do not describe interventions here. | **Guidelines Palliative care**  - WEBSITE: Pallialine <https://palliaweb.nl/richtlijnen-palliatieve-zorg/richtlijn>  - APP: Palliarts <https://palliaweb.nl/richtlijnen-palliatieve-zorg/hulpmiddelen/palliarts>  - WEBSITE: Palliaguide: <https://palliaguide.nl/#!/projects/2/tree/112>  - Summary cards (Also accessible via the app) <https://shop.iknl.nl/search/samenvattingskaart/>  **National Palliative Care Consultation Service:**  The telephone consultation service for palliative care is available nationwide 24/7. There is one national phone number (088-6051444) that, based on the caller’s postal code, connects to the appropriate consultation team. |
| 3) Formulating the goal of the care plan | Goal care plan | For each prioritized symptom or concern, formulate a specific care goal. Be as concrete as possible. The goal should align with what the patient experiences, wishes for, and considers important. |  |
| 4) Draw up a proactive care plan | Proactive care plan | Develop the care plan based on what the patient still wants and what is feasible, meaningful, and appropriate. Also, consider a next step in case the intervention does not have the desired effect. | **Advanced Care Planning**  Thematic page Advance Care Planning (Palliaweb, 2021) <https://palliaweb.nl/zorgpraktijk/proactieve-zorgplanning> |
|  | Non-pharmacological Interventions | - Use guidelines - Tailor interventions to specific wishes and coping mechanisms of the patient - Actively involve relatives | **Guidelines palliative care (see above)** |
|  | Pharmacological Interventions | - Use guidelines |  |
| 5) Evaluation | Making agreements about evaluation per intervention | Make the following agreements per intervention:   - Next evaluation moment - Evaluation by - How to measure the effect (measurement tool) |  |
|  | Evaluate | Measure the effect by symptom severity, experience, patient functioning and wellbeing/comfort   - Involve the patient and relatives in the evaluation   Document the outcome measure and the process evaluation in the patient record |  |
| **STAP 4 – Adapt care plan as needed and constant evaluate** | Evaluate the effect | Evaluate the effect of the implemented interventions:  **Effect good**  Continue to evaluate (STEP 3)   - Be aware that underlying causes may change over time. - Keep in mind that some symptoms cannot be fully taken away   **Effect absent/limited**  Adapt the care plan (STEP 2) OR  Re-analyze the problem (STEP 1) OR  Accept the situation |  |
| **At each step** | Coordinate with the patient and their loved ones  Consider using measurement instruments  Think proactively about the future  Ensure proper documentation and good handover | | |
