# Supplementary material for: Implementing palliative reasoning in multiprofessional palliative care: A participatory action research in the Netherlands
Source: PLoS One. 2026 Aug 28;21(8):e0354089. doi: 10.1371/journal.pone.0354089 (PMC13524244; doi:10.1371/journal.pone.0354089)
Supplement: Supplementary file 2 [file pone.0354089.s002.docx]

| **Cluster** | **Name** | **Strategy specification** | | | | | | |
| --- | --- | --- | --- | --- | --- | --- | --- | --- |
|  |  | **Actor** | **Action** | **Target of the action** | **Temporality** | **Dose** | **Outcomes affected** | **Justification** |
| **Use of Evaluative and iterative strategies** | Assess for readiness and identify barriers and facilitators | Champions/coaches together with  HCPs | Assessment of barriers and facilitators and how to deal with them.  Example:  - First by champions/coaches and evaluated by HCPs/manager(s)  - Survey under HCPs/managers  - Team meeting. If evaluated in a meeting this could be merged with the meeting on reporting in the electronic health record. | To determine HCPs degree of readiness to implement, barriers that may impede implementation, and strengths that can be used in the implementation effort | Before the start of the implementation | One time | Implementation  Acceptability | Ensures to tailor implementation strategies to local needs/context |
|  | Purposefully reexamine the implementation | Champions/  coaches together with HCPs | Organize meetings with HCPs to reflect and evaluate on the implementation process | To monitor progress and adjust clinical practices and implementation strategies to continuously improve the quality of care. | Throughout implementation | After each step of implementation  (2-3x, 1-2h) | Implementation  Acceptability | Ensures to tailor implementation strategies to local needs/context |
|  | Conduct cyclical small tests of change | Champions/  Coaches together with HCPs | Implement PR in several steps.  Start by every new implementation step with a meeting with all stakeholders to discuss new implementation steps, the process, responsibilities and roles. These meetings could be merged with reflection meetings. | To support HCPs in getting to know and master various aspects of PR and to understand the coherence of all aspects.  To evaluate change within the setting/HCPs, to study changes for insights on how to do better, and to refine implementation process if needed. | Throughout implementation | Several cycles of tests of change (2-3x, 1-2h) | Implementation  Acceptability  Fidelity | Ensures to tailor implementation strategies to local needs/context |
| **Provide interactive assistance** | Provide local technical assistance | Input: champions/coaches together with HCPs  Carrying it out: ICT personal | Documentation must be a part of implementation. Look at the electronic health record system prior to implementation and discuss with all HCPs the following:  - where is which patient information located  - where will patient information gather through PR be reported in system  - Are these locations useful or do they have to be adapted  - implementation of PROMS  - discuss options with ICT to adapt the electronic health record system (outline in line with PR, add PROMS).  Example:  - First investigated by champions/coaches and evaluated by HCPs  - Team meeting  - If evaluated in a meeting this could be merged with the meeting on the assessment of barriers and facilitators.  Developed information standard for electronic health records could help. | To fit PR with existing infrastructure (reporting in electronic health record) and make PR more compatible. | Before the start of the implementation | One time | Implementation  Feasibility  Sustainability | Supports information technology infrastructure |
| **Adapt and tailor to context** | Tailor strategies | Champions/coaches | Study all proposed implementation strategies and decide which ones to use in own implementation. Also, a possibility to add own strategies based on barriers and facilitators identified within own context. | To address barriers and leverage facilitators that were identified through earlier data collection. | Before the start of the implementation | One time | Implementation  Feasibility | Ensures to tailor implementation strategies to local needs/context |
|  | Promote adaptability | Champions/coaches | Investigate the fit between the content and context and make agreements about the implementation, content, strategies and responsibilities and roles.  - Investigate the content of the cyclical education steps, when to implement what en how  - Investigate all available educational material and decide which ones are useful for the own context or might be adapted.  - Investigate how to give form to learning collaboratives | To meet local needs and clarify which elements of the innovation must be maintained to preserve fidelity. | Before the start of the implementation | One time | Implementation  Feasibility  Fidelity | Ensures to tailor implementation and innovation to local needs/context |
| **Develop stakeholder interrelationships** | Identify and prepare champions | Manager(s) | Identify and prepare champions within the organization and create physical opportunity (hours) for them to fulfil their role.  Champions = opinion leaders/implementation facilitators  (HCPs with informal influence on the attitudes and behaviors of others and with subject matter expertise who assist, coach, or support implementation)  Example: Nursing staff/physician  Use identified characteristics applicable to the role (COM-B):  Physical capability  - Having the skill to organize the implementation (organizational tasks)  Psychological capability  - Understanding of PC  - Understanding of the connections between PR and PC  - Understanding of PR, PC with regular care in NH/primary care  - Leadership skills  - Communication skills  Social opportunity  - Have informal power within the team and organization  - Have connection with team and organization  Automatic motivation  - Be open to PR, want to learn new things and, above all, reach others with your own motivation  - Have an affinity with PC  Reflective motivation  - Intending to restructure environment, integrate PR into work process | To have HCPs that dedicate themselves to supporting, marketing, and driving through an implementation, overcoming indifference or resistance that the intervention may provoke in an organization | Before the start of the implementation | One time | Implementation  Adoption | Increases psychological capability of champions (COM-B)  Increases reflective motivation of champions and HCPs (COM-B) |
|  | Inform local opinion leaders | Champions/coaches | Make sure that all stakeholders within the organization/team are sufficiently informed about the implementation. Regularly inform stakeholders about the implementation that are not directly participating. | To involve all stakeholders with sufficient power in the organization, to be on one line and to hope for their support to influence colleagues to adopt it. | Before the start of the implementation  Throughout the implementation | Several times  (can be short conversations) | Implementation  Adoption  Sustainability | Increased psychological capability of other stakeholders (COM-B)  Supports information technology infrastructure |
|  | Involve executive boards | Manager(s) | Make sure all existing governing structures within an organization are involved and regularly updated about the implementation processes. | To involve all stakeholders within an organization | Before the start of the implementation  Throughout the implementation | Several times  (Can be short conversations/  newsletters) | Implementation  Adoption  Sustainability | Supports information technology infrastructure |
| **Train and educate stakeholders** | Develop educational materials | Champions/coaches | Education material was developed* and is available for free [see website PR] **.  All materials were tailored to the local context before use. | To tailor educational material to context and involved HCPs | Before the start of the implementation | One time | Implementation  Acceptability | Increases psychological capability of HCPs (COM-B)  Ensures to tailor implementation strategies to local needs/context |
|  | Distribute educational materials | Champions/coaches | A list of supportive sources/tools is freely available within the practical elaboration of PR. The practical elaboration can be downloaded and distributed within all stakeholders involved (managers, champions, coaches, HCPs). [see website PR] **. | To support HCPs in mastering PR. | Before the start of the implementation | One time | Implementation  Adoption | Increases psychological capability of HCPs (COM-B)  Ensures to tailor implementation strategies to local needs/context |
|  | Use train-the-trainer strategies | Manager(s): identification  Coaches: Train-the-trainer (train the champions): | Identify local experts that are able to coach the local champions/support HCPs and create physical opportunity (hours) for them to fulfil their role.  Experts = Implementation facilitators  (HCPs with subject matter expertise who coach implementation)  - HCPs with an already existing expertise/overarching role in the organization  - HCPs that could fulfil such an overarching role  (example: nurses specialized in PC)  Use identified characteristics applicable to the role (COM-B):  Psychological capability  - Experts in PC  - Understanding the local context  - Being able to empathize with champions/HCPs  - Leadership skills  - Communication skills  Social opportunity  - Have informal power within the team and organization  - Have connection with team and organization  Reflective motivation  - Intending to restructure environment, integrate PR in workproces | To support implementation process, champions, HCPs and to sustain implementation. | Before the start of the implementation | One time | Implementation  Adoption  Sustainability  HCPs  Leadership development (champions) | Increases psychological capability of champions and HCPs (COM-B)  Increases reflective motivation of champions and HCPs (COM-B) |
|  | Conduct educational meetings | Champions/coaches | Engage in conversation with each other (managers/HCPs, champions, coaches), that everyone involved perceives the need to change and to assume their role. | That all stakeholders perceive the same tension for change, understand each other and perceive the same goals of implementation. | At the start of the implementation  Throughout the implementation if necessary | One time/several times (can be informal conversations) | Implementation  Adoption  HCPs  Motivation | Supports psychological capability of HCPs, managers (COM-B)  Supports reflective motivation of champions, coaches, HCPs, managers (COM-B) |
|  |  |  | Provide education for HCPs.  - Principles of palliative care  - Identification of patients with palliative care needs  - ROMS and their use  - Communication with patients and relatives | To support HCPs in mastering PR. | At the start of the education  During implementation | Depending on what is necessary, one lesson 1-2h | HCPs  Knowledge  Motivation  Patient-centeredness | Supports psychological capability of HCPs (COM-B) |
|  | Create a learning collaborative | Champions/coaches | Format a group(s) of HCPs within the organization in which PR will be implemented (ward/district team)  Facilitate and organize patient discussions for these groups involved, in which PR will be used. | To implement PR in practice/workflows of HCPs, support HCPs in mastering PR in practice, to learn from each other. | Before the start of the implementation  Throughout the implementation | Patient discussion every 1-3 weeks, depending on context and possibilities.  (each discussion: 30min-1h) | Implementation  Adoption  Sustainability  HCPs  Emancipation (nursing staff) | Supports psychological capability of HCPs, champions, coaches (COM-B)  Supports reflective motivation of HCPs (COM-B)  Supports social and physical opportunity of HCPs (COM-B) |
| **Utilize financial strategies** | Access new funding | Manager(s) | Look into possible implementation fees to facilitate implementation, if internal financial support is scarce (support extra time/hours needed for implementation: champions, coaches, HCPs) | To support organization in facilitating meetings, implementation, and champions, coaches, HCPs in their roles. | Before the start of the implementation | One time | Implementation  Adoption  Sustainability  HCPs  Motivation | Supports physical opportunity of managers, HCPs, champions, coaches.  Supports financial shortcomings |
| **Change infrastructure** | Mandate change | Champions/coaches | Include managers in meetings to declare priority and determination, if necessary, also throughout the project. | To show HCPs, champions, coaches, that the organization (manager) is determined to have PR implemented and committed. | Throughout the implementation | Several times | Implementation  Adoption  HCPs  Motivation | Supports reflective motivation of HCPs, champions, coaches (COM-B) |
|  | Change accreditation | Manager(s)/champions/  coaches | Build accreditation system (intern/extern) | To encourage the use of PR and its implementation strategies. | Before the start of the implementation | One time | HCPs  Motivation | Supports reflective motivation of HCPs (COM-B) |
| * Teaching material: Basic education PR, Basic education PROMS, Example material reflection meetings; Manual for chairperson of multiprofessional patient discussions where PR is implemented; Worksheet to fill in patient information, who is discussed in multiprofessional patient discussion; Manual USD-4d (PROM); Patient information letter USD-4d  ** https://palliaweb.nl/zorgpraktijk/palliatief-redeneren | | | | | | | | |
